# Home grocery delivery improves the household food environments of behavioral weight loss participants: Results of an 8-week pilot study

**DOI:** 10.1186/1479-5868-4-58

**Published:** 2007-11-14

**Authors:** Amy A Gorin, Hollie A Raynor, Heather M Niemeier, Rena R Wing

**Affiliations:** 1 Department of Psychology, University of Connecticut, 406 Babbidge Road, Unit 1020, Storrs, CT 06269-1020, USA; 2 Department of Nutrition, University of Tennessee, 1215 W. Cumberland Ave, Knoxville, TN 37996-1920, USA; 3 Brown Medical School, The Miriam Hospital, Weight Control and Diabetes, Research Center, 196 Richmond Street, Providence, RI 02903, USA

## Abstract

**Background:**

Household food availability is consistently linked to dietary intake; yet behavioral weight control treatment includes only minimal instruction on how to change the home environment to support dietary goals. This pilot study examined whether it is feasible to change the household food environments of behavioral weight loss participants through the use of a commercially available grocery home delivery service.

**Methods:**

Overweight participants (N = 28; BMI = 31.7 ± 3.6 kg/m^2^; 89.3% women, 47.9 ± 9.5 years) were randomly assigned to 8-weeks of standard behavioral weight loss (SBT) or to SBT plus home food delivery (SBT+Home). SBT+Home participants were instructed to do their household grocery shopping via an online service affiliated with a regional supermarket chain and were reimbursed for delivery charges.

**Results:**

Compared to SBT, SBT+Home produced significantly greater reductions in the total number of foods in the home (p = .01) and number of foods that were high in fat (p = .002). While the groups did not differ in 8-week weight losses, within SBT+Home there was a trend for the number of home deliveries to be associated with weight loss (p = .08). Participants reported that the home delivery service was easy to use and that it helped decrease impulse purchases and lead to healthier choices; however, few planned to continue using the service after the study.

**Conclusion:**

Encouraging weight loss participants to use a commercially available online grocery ordering and home delivery service reduces the overall number of food items in the home and decreases access to high-fat food choices. More research is needed to determine whether this is a viable strategy to strengthen stimulus control and improve weight loss outcomes.

## Background

Several studies suggest that eating choices are influenced by food cues in the home environment [1-4]. For example, Campbell and colleagues recently demonstrated that having unhealthy foods available in the home is linked to intake of these types of foods [5]. Similar relationships have been reported regarding household availability and intake of high-fat foods [6] and fruits and vegetables [7]. The behavioral model that forms the theoretical basis of behavioral weight control treatment recognizes the importance of environmental cues in determining eating and exercise behaviors [8]. Weight loss participants are taught the concept of stimulus control and are encouraged to change their homes to support their new healthy behavioral goals [9]. However, only 1–2 sessions in a typical 6 month program are dedicated to stimulus control and minimal instruction is given to participants about how to manipulate their home environments to promote weight loss.

Efforts to strengthen stimulus control through strategies such as food provision have demonstrated success in improving weight loss outcomes [10-12]. In fact, many commercial weight loss programs (e.g., Jenny Craig, NutriSystem) provide food to participants as a central part of their treatment plan. To date, these direct manipulations of eating cues have focused on the foods provided for the individual weight loss participant's consumption and not their overall household food environments. By focusing on the individual alone, it is likely that participants will continue to grocery shop for other family members and/or be surrounded by foods in the home that may not be consistent with their dietary goals. New strategies are needed to change the overall household food environments of weight loss participants to support healthy eating choices.

The goal of this pilot study was to examine the feasibility of changing the household food environments of behavioral weight loss participants through the use of a commercially available grocery home delivery service. We examined both the impact and acceptability of the service compared to standard behavioral weight loss treatment over an 8-week period.

## Methods

### Participants

Twenty-eight overweight individuals were recruited from advertisements in local newspapers. To be eligible, participants had to be 21–65 years old, have a BMI ≥ 25 kg/m^2^, and live in an area where the delivery service was available (determined by zip code). Exclusionary criteria included having a physical or psychiatric disorder that would affect retention or the safety of the intervention; pregnant or nursing within the past 6 months or plans to become pregnant during the study period; currently participating in another weight loss program; or currently taking medications that could affect weight. All participants provided written consent and the study protocol was reviewed and approved by The Miriam Hospital.

### Interventions

Participants were randomly assigned to standard behavioral weight loss treatment (SBT) or to standard behavioral weight loss treatment plus home food delivery (SBT+Home). Both groups received 8-weeks of SBT focused on behavioral and cognitive skills. Groups met weekly for 60 minutes, led by doctoral level therapists with backgrounds in nutrition and/or behavioral psychology and prior experience conducting weight loss treatment. All participants were prescribed a low-calorie (1200–1500 kcal/day, depending on initial body weight) and low-fat (≤ 30% of daily calories) diet and were instructed to increase their exercise to 150 minutes per week. Participants self-monitored their caloric intake and exercise daily and received written feedback from the interventionists. Lesson topics included goal setting, problem solving, stimulus control, cognitive restructuring, and relapse prevention.

#### Additional treatment components specific to SBT+Home

Throughout the 8-week program, participants in the SBT+Home condition were encouraged to order all of their groceries through an online service affiliated with a regional supermarket chain. Participants were required to pay for their own groceries, but the study reimbursed the delivery costs ($3.95–$7.95 per delivery, depending on the size of the order). To assist in the use of the service, SBT+Home participants attended a 30 minute individual session with a nutritionist prior to the start of the 8-week program. During this session, which focused exclusively on the use of the online ordering service, the nutritionist led the participant through a practice order online, highlighted features such as searching for desired items by nutritional content, and answered any questions about how to use the service. The nutritionist also assessed whether the participant had access to a computer to place their orders. Free computer access was offered at the clinic; however, none of the participants utilized this option. The individual training session and computer access were not offered to SBT participants.

### Assessments

Participants were assessed at baseline and at the conclusion of the 8-week program.

### Demographics

Basic demographic variables were assessed by self-report at baseline. Participants were also asked to indicate whether they had used the home delivery service before, and if so, how many times.

### Weight

Weight was measured at baseline and follow-up on a calibrated scale. Height was measured at baseline using a wall mounted stadiometer.

### Home food environment

A questionnaire to assess household food availability was modified from Raynor et al. [6] based upon the 60-item Block Food Frequency Questionnaire [13]. The inventory has 26 common food categories that represent items either very high in fat content (> 45% energy from fat – 14 items) or low in fat content (< 18% energy from fat – 12 items) (see Table 1 for complete list). The inventory includes high- and low-fat items because these are the foods that behavioral weight control participants are encouraged to change in their homes to promote adherence to the low-calorie, low-fat dietary prescription. The items do not include staples, such as breads and rice, because the availability of these types of foods is not anticipated to change as a result of weight loss treatment. Participants indicated whether or not each food category was currently available in their home regardless of amount. To increase the accuracy of reports, participants completed this measure at home and were instructed to check all places where food might be stored including cabinets, pantries, and refrigerators. A prior version of this inventory was used in a study by Raynor et al. [13] establishing strong two-week test-retest reliability for high-fat and low-fat items (p's<.001) as well as an association with high-fat items and dietary fat intake (p < .001). Using the current version of the inventory, we have also found strong agreement between the ratings of spouses living in the same house [14]. For the present study, we calculated the total number of food categories available in the home (out of 26 possible food categories) as well as the total number of high-fat (possible total of 14 food categories) and low-fat food categories (possible total of 12 food categories).

**Table 1:** Items on the household food inventory

**High-fat food categories (>45% energy from fat)**	**Low-fat food categories (< 18% energy from fat)**
Butter or margarine	Apples, applesauce, pears
Mayonnaise	Cantaloupe, mango, papaya
Bacon, sausage, other breakfast meat	Oranges, grapefruit, tangerines
Hot dogs, bologna, lunch meat	Orange juice or grapefruit juice
Cream, whipped cream	Other fruit juices, fortified fruit drinks
Nuts or peanut butter	Tomatoes, tomato juice, pico de gallo
Cheese	Broccoli
Beef, pork, lamb	Spinach
Potato chips, corn chips, tortilla chips	Mustard greens, turnips greens, collards, kale
Cookies	Carrots, mixed vegetables containing carrots
Cakes, pies	Lettuce
Pastry such as doughnuts or sweet rolls	Sweet potatoes, yams
Whole milk	
High-fat frozen desserts such as ice cream	

#### Ratings of the home delivery service

SBT+Home participants also completed a 9-item questionnaire assessing both the impact (e.g., "Shopping on-line decreased the amount and variety of unhealthy food items in my house") and the acceptability (e.g., "I found the home delivery service easy to use") of the home delivery service.

### Statistical Analyses

Data were analyzed using SPSS for Windows version 14.0. Descriptive statistics are presented as means ± standard deviation (SD) for continuous variables or percentages for categorical variables. Changes in home food availability and weight were examined with repeated measures analyses of variance. Within SBT+Home, the relationship between changes in weight and number of home deliveries was examined with a partial correlation, controlling for baseline weight.

## Results

There were no baseline demographic differences between SBT (n = 15) and SBT+Home (n = 13) participants (Table 2). The mean age of the sample was 47.9 ± 9.5 years, with a BMI of 31.7 ± 3.6 kg/m^2^. The majority of the sample was female (89.3%), Caucasian (89.3%) and married (60.7%). None of the participants had used the home delivery service prior to study entry. Follow-up weight data were available on 14 (93%) SBT participants and 11 (85%) SBT+Home participants and questionnaire data was available on 15 (100%) SBT participants and 9 (70%) SBT+Home participants. Data from treatment completers are presented below.

**Table 2:** Baseline characteristics by treatment condition

	**SBT **(n = 15)	**SBT+Home **(n = 13)
**Age**	48.2 ± 11.4	47.5 ± 7.2
**Gender (% female)**	87%	92%
**Marital status (% married)**	60%	62%
**Ethnicity (% Caucasian)**	87%	92%
**Weight (lbs)**	187.2 ± 31.7	185.7 ± 27.9
**Body mass index**	32.3 ± 3.8	31.3 ± 3.3

Note: There were no significant differences between treatment conditions on any of the above variables.

### Impact of using the home delivery service

SBT+Home participants received an average of 4.1 ± 1.8 home deliveries (range 1–7) over the 8-weeks of the study. As seen in Table 3, SBT+Home produced significantly greater reductions in the total number of food categories available in the home than SBT (p = .01). This was driven by a greater decrease in the number of high-fat foods in SBT+Home compared to SBT (p = .002). There was no main effect of time, group, or group × time interaction for the number of low-fat foods available in the home.

**Table 3:** Means and standard deviations of home environment variables at baseline and post-treatment

		**SBT**	**SBT+Home**	**p value**		
				
				**Group**	**Time**	**Group × Time**
**Total Number of Foods in the Home**	BL	14.57 ± 2.98	16.11 ± 3.22	.858	.006	.010
	Follow-up	14.43 ± 2.79	12.44 ± 4.16			

**Number of High-Fat Foods in the Home**	BL	8.07 ± 2.15	9.11 ± 2.42	.768	.000	.002
	Follow-up	7.27 ± 2.05	5.67 ± 3.12			

**Number of Low-Fat Foods in the Home**	BL	6.64 ± 1.91	7.00 ± 2.60	.875	.607	.332
	Follow-up	7.36 ± 1.86	6.78 ± 1.48			

A significant main effect of time was observed for weight loss, with average losses of 8.4 ± 7.4 lbs across the 8-weeks, F(1, 23) = 29.5, p < .001. There was no difference in weight loss between the two conditions (7.5 ± 8.5 vs. 9.0 ± 6.7 lbs in SBT+Home and SBT, respectively, p = .64); however, within SBT+Home, there was a trend for the number of home deliveries to be correlated with weight loss, r = .71, p = .08.

### Acceptability of using the home delivery service

SBT+Home participants endorsed that the home delivery service helped them decrease the amount of unhealthy food in their home, decrease impulse purchases, and make healthier choices (Table 4). In general, they reported that the home delivery service was easy to use and that they were satisfied with customer service. Seventy-five percent endorsed they had recommended the service to others. However, less favorable ratings were given concerning the prices of foods, the availability of desired items, and plans to continue using the service at the conclusion of the study.

**Table 4:** Home delivery impact and acceptability ratings in SBT+Home participants

	M ± SD
Shopping on-line decreased the amount and variety of unhealthy food items in my house	5.1 ± 1.6
Shopping on-line decreased my impulse food purchases	5.4 ± 1.4
Using the home delivery service helped me make healthier food choices	5.5 ± 1.9
I found the home delivery service easy to use	5.3 ± 2.1
I plan to continue using the home delivery service now that the study is over	3.6 ± 2.1
I was satisfied with the home delivery service's customer service (i.e receiving correct items)	5.6 ± 1.3
The home delivery service usually had the specific items I wanted	4.3 ± 4.5
Prices on the home delivery service were reasonable and comparable to other grocery stores	4.3 ± 1.5
Have you recommended the home delivery service to anyone? (% yes)	75%

Note: Items 1–8 rated on a 7-point Likert scale, with 1 = strongly disagree and 7 = strongly agree

## Discussion

This pilot study demonstrated that encouraging behavioral weight loss participants to order their groceries online and have their food delivered to their home decreased the total number of foods in their home and more specifically, the availability of high-fat foods. While we did not find differential weight losses between the home delivery condition and the standard behavioral weight loss treatment condition over this short time period, in the home delivery condition there was a trend for weight loss to be associated with the number of home deliveries made during the 8 week program. The results suggest that the use of a commercially available online shopping and home delivery service may be an effective way to strengthen stimulus control and modify the household food environments of weight loss program participants.

Effective models for changing the home food environment exist [12,15]; for example, Ebbeling and colleagues [15] demonstrated that delivering non-caloric beverages to the homes of adolescents decreased intake of sugar sweetened beverages by 82% over a 25 week period resulting in less weight gain in the heaviest adolescents compared to the control group. The present study adds to this literature by suggesting that the total food environment, not just availability of beverages, can be positively changed by encouraging households to have healthy foods delivered to their homes.

Participants reported many positive benefits of ordering their groceries online including making healthier choices while shopping and making fewer impulse purchases. The online service allowed people to view and reuse prior grocery lists, which may have helped improve the household food environment by providing more structure to meal planning and food purchases. Despite these benefits, acceptability of the service was low. Participants reported that they did not plan to continue using the home delivery service after the conclusion of the study, perhaps because they reported being only moderately satisfied with the prices of food available. The greater attrition in the home delivery condition may have also reflected reluctance to order groceries online. It is also important to note that computer access was offered to participants during the study, although no one used it, and the deliveries fees were reimbursed. Computer access and delivery fees might both be significant barriers to widespread adoption of this type of service, particularly in lower socioeconomic status populations that have more limited access to the internet [16]. Further research is needed to explore barriers to continued use of commercially available home delivery services and to identify subgroups for whom this strategy might be most effective, such as weight loss participants who have limited time for grocery shopping and as a result rely heavily on fast food and prepared meals.

Limitations of the present study include the small sample size, short follow-up period, and the absence of dietary intake and physical activity measures. By encouraging participants to order their food online and have it delivered to their home rather than go to the grocery store, the home delivery condition may have inadvertently decreased energy expenditure in some participants. Also, since participants in the home delivery condition also attended a brief session with a nutritionist, it is possible that this session and not the subsequent use of the service itself, led to decreases in high-fat foods in home. Given that the session focused exclusively on use of the online service and did not include more general dietary advice, this possibility is unlikely but cannot be directly tested within the study design. More work is needed to determine the long-term impact of using an online grocery ordering and home delivery service as part of behavioral weight loss treatment and the types of participants for whom it is best suited.

## Conclusion

This initial pilot study suggests that encouraging weight loss participants to order their groceries online and have them delivered to their homes may help decrease cues for unhealthy eating within the home environment.

## Competing interests

The author(s) declare that they have no competing interests.

## Authors' contributions

AG, HR, and RW designed the study. AG, HR, and HN delivered the intervention. AG performed the statistical analyses and drafted the manuscript. AG, HR, HN, and RW revised the manuscript and all authors approved the final version.
